# Heterogeneous shedding of influenza by human subjects and its implications for epidemiology and control

**DOI:** 10.1038/srep38749

**Published:** 2016-12-14

**Authors:** Laetitia Canini, Mark E. J. Woolhouse, Taronna R. Maines, Fabrice Carrat

**Affiliations:** 1 Centre for Immunity, Infection & Evolution, University of Edinburgh, UK; 2 Influenza Division, National Center for Immunization and Respiratory Disease, Centers for Disease Control and Prevention, Atlanta, Georgia, USA; 3 INSERM, UMR_S 1136, Institut Pierre Louis d’Epidémiologie et de Santé Publique, Paris, France; 4 Sorbonne Universités, UPMC Univ Paris 06, UMR_S 1136, Institut Pierre Louis d’Epidémiologie et de Santé Publique, Paris, France; 5 Assistance Publique Hôpitaux de Paris, Hôpital Saint Antoine, Paris, France

## Abstract

Heterogeneity of infectiousness is an important feature of the spread of many infections, with implications for disease dynamics and control, but its relevance to human influenza virus is still unclear. For a transmission event to occur, an infected individual needs to release infectious particles via respiratory symptoms. Key factors to take into account are virus dynamics, particle release in relation to respiratory symptoms, the amount of virus shed and, importantly, how these vary between infected individuals. A quantitative understanding of the process of influenza transmission is relevant to designing effective mitigation measures. Here we develop an influenza infection dynamics model fitted to virological, systemic and respiratory symptoms to investigate how within-host dynamics relates to infectiousness. We show that influenza virus shedding is highly heterogeneous between subjects. From analysis of data on experimental infections, we find that a small proportion (<20%) of influenza infected individuals are responsible for the production of 95% of infectious particles. Our work supports targeting mitigation measures at most infectious subjects to efficiently reduce transmission. The effectiveness of public health interventions targeted at highly infectious individuals would depend on accurate identification of these subjects and on how quickly control measures can be applied.

Heterogeneity of infectiousness is an important feature of the spread of many infections, such as *E. coli* O157, paratuberculosis or salmonella and has been shown to have implications for disease dynamics and control^1^. Lau *et al*. showed evidence of heterogeneity in viral shedding in symptomatic patients naturally infected by influenza A virus^2^. However, they could not document the overall time course of the infection as the time of exposure is unknown for naturally infected cases, so the implications of heterogeneous infectiousness for the epidemiology and control of influenza remain unclear.

For a transmission event to occur, an influenza infected individual needs to release infectious particles via respiratory symptoms. Key factors to take into account are virus dynamics, virus spreading in relation to respiratory symptoms, the amount of virus shed and, importantly, how these vary between infected individuals^3^,^4^. Understanding influenza transmission process and the factors associated with increased infectiousness is relevant to the implementation of effective mitigation measures^5^.

Influenza within-host dynamics modelling allows a quantitative description of the infection and/or symptoms dynamics and has received increasing attention over the past decade^6^,^7^,^8^,^9^,^10^,^11^,^12^,^13^,^14^. We extend a previously published model describing viral kinetics (VK) and symptoms dynamics (SD)^6^ to include the dynamics of respiratory symptoms. The model is fitted to virological, systemic and respiratory symptoms and is used to investigate how within-host dynamics relates to infectiousness.

Using a mathematical modelling approach, we aim to link viral shedding to influenza within-host dynamics and to identify parameters associated with heterogeneity of infectiousness.

## Material and Methods

### Data

We used data from the control arm of five studies conducted between 1993 and 1997 comprising 56 healthy volunteers experimentally infected with influenza virus. These studies were randomized, double-blind, placebo-controlled registration studies of zanamivir treatment of H1N1 influenza virus (NAIA1001, NAIA1002, NAIA1003, NAIA1004 and NAIA1010). All were approved by ethics committees, and the volunteers gave their written informed consent. Volunteers were eligible for these studies if they were Caucasian men or women aged from 18 to 40 years, with serum hemagglutinin antibody titres of <1:8 to the relevant virus strains. They were non-smokers or smoked an average of less than 10 cigarettes per day and agreed not to smoke for the duration of the isolation period. They were judged to be healthy based on medical records, physical examination and laboratory investigations^6^.

The volunteers under placebo were challenged at 08:00 with 10^5^ median tissue culture infective doses (TCID_50_) of influenza A/Texas/91/36 (H1N1) virus intranasally and were monitored daily for the following 7 or 8 days. A sample for viral shedding kinetics analysis was taken from each volunteer 8 or 9 times. In four studies (48 subjects), sample collection took place at 08:00 on day 0 (D0) before the challenge, and then on D1, D2, D3, D4, D5, D6, D7, and D8; no D8 sample was collected in the fifth study (8 subjects). The following symptoms were noted: earache, runny nose, sore throat, coughing, sneezing, breathing difficulties, muscle ache, fatigue, headache, feverish feeling, hoarseness, and chest discomfort. The intensity of each symptom was scored by the patient from 0 (none) to 3 (severe). Symptoms data were collected twice a day, at 08:00 and 20:00 on the same days as the viral titre samples^6^.

We used the systemic symptoms score constructed in a previous study (i.e. the sum of the scores for feverish feeling, headache, fatigue and muscular fever)^6^. We built similarly a respiratory symptom score by summing the scores for runny nose, sore throat, coughing and sneezing. This led to symptoms scores ranging between 0 and 12. We focused on these symptoms as they were the most frequent and also the most likely to spread virus in the environment of the infected subjects.

### Model

We adapted the within-host dynamic model previously developed by Canini and Carrat^6^ to take into account respiratory symptoms dynamics. This model described influenza viral kinetics as:


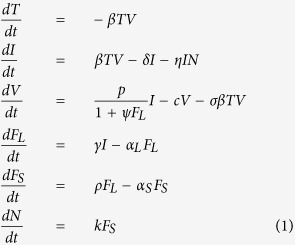


**Figure 1:**
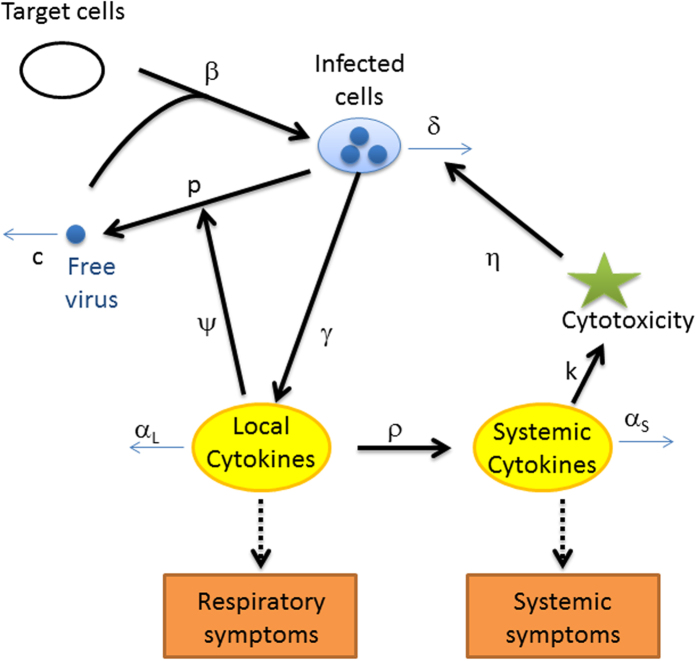
Schematics of the within-host dynamic model of influenza infection. Infectious viruses, *V*, infect target cells, *T*, at rate *β* and are cleared at rate *c*. The infected cells, *I*, produce virus at rate *p* and are lost at rate *δ*. The infected cells induce the production of local pro-inflammatory cytokines, *F*_*L*_, at rate *γ*. The local pro-inflammatory cytokines decrease *p* by a factor *ψ*, are cleared at rate *α*_*L*_ and induce the production of systemic pro-inflammatory cytokines, *F*_*S*_, at rate *α*. Systemic pro-inflammatory cytokines are cleared at rate *α*_*S*_ and induce the activation of the cytotoxic activity, *N*, at rate *k*. Cytotoxic activity removes infected cells at rate *η*. The respiratory and systemic symptoms were used as proxy for *F*_*L*_ and *F*_*S*_, respectively.

In this model as represented in Fig. 1, infectious viruses, *V*, infect target cells, *T*, at rate *β* and are cleared at rate *c*. The infected cells, *I*, produce virus at rate *p* and are lost at rate *δ*. The infected cells induce the production of local pro-inflammatory cytokines, *F*_*L*_, at rate *γ*. The local pro-inflammatory cytokines decrease the virus production rate, *p*, with an effect *ψ*, are cleared at rate *α*_*L*_ and induce the production of systemic pro-inflammatory cytokines, *F*_*S*_, at rate *ρ*. As the cytokines levels were not measured, we set their dimension to F. Systemic pro-inflammatory cytokines are cleared at rate *α*_*S*_ and induce the activation of the cytotoxic activity, *N*. Cytotoxic activity, whose dimension was set to cell^−1^.d^−1^, is induced at rate *k* by systemic pro-inflammatory cytokines and removes infected cells at rate *η*. We set *k* to 1 F^−1^. cell^−1^, d^−2^, without loss of generality, as this changes only the units in which cytotoxic activity is measured. A conversion factor *σ* is needed for the conversion from infectious virus, *V* in our model, to TCID_50_/mL as measured by the experimental data^15^. Here we set *σ* = 0.69 TCID_50_/mL. cell^−1^ which is consistent with 1 mL virus stock having half the number of plaque forming units (PFUs) as the TCID_50_. The detailed dimension analysis is shown in supplementary material.

The initial values for the different compartments were: *T*_*0*_ = 4×10^8^ cells^16^, *I*_*0*_ = *F*_*L,0*_ = *F*_*S,0*_ = *N*_*0*_ = 0 as we assumed that there were no infected cells and the innate immune response (i.e. systemic and local pro-inflammatory cytokines, and cytotoxic activity) was not activated. T_0_ was determined by Baccam *et al*. as the number of epithelial cells lining the nasal turbinates of the respiratory tract^16^. V_0_ was estimated as a parameter. We did not assume that V_0_ was the inoculated dose as we have no information concerning the quantity of virus remaining in the nose.

Local and systemic pro-inflammatory cytokines levels, particularly IFN-*α* and IL-6 correlate with respiratory and systemic symptom dynamics^17^,^18^,^19^,^20^,^21^. We therefore decided to use the symptom scores as surrogate for pro-inflammatory cytokine levels. The respiratory symptoms were modelled as:


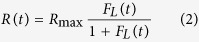


*R(t)* represents the respiratory symptom score at time *t* and *R*_*max*_ = 12 is the upper limit of the respiratory symptom score scale. We computed the systemic symptom score in a similar fashion, as a function of the level of systemic cytokines *F*_*S*_*(t)* and the upper limit of the systemic symptom score scale, *S*_*max*_ = 12.

### Parameter estimation

Nonlinear mixed-effect models were used in order to take into account the inter-individual variability (IIV) and to compute individual parameters (see Supplementary Material). Each parameter is described by a fixed effect determining the average value in the population and a random effect determining the IIV. Additionally, we estimated the full variance-covariance matrix for random effect and used the normalized covariance to describe the correlations between the parameters distributions. Population parameters were estimated by likelihood maximization using the stochastic approximation of the expectation-maximization (SAEM) algorithm, implemented in MONOLIX 4.3.2 (http://www.lixoft.eu/). Individual parameters are computed as empirical Bayes estimates. We simultaneously fitted the viral titre data, the systemic symptom score data and the respiratory symptom score data.

When testing different models, we chose the model providing the smallest Bayesian information criteria (BIC)^22^. We performed a practical identifiability analysis as suggested by Brun *et al*.^22^, using the package FME implemented in R^23^.

### Natural history parameters

We computed the population and individual infectiousness and other epidemiological parameters (incubation period and duration of symptomatic phase) to predict how these parameters would vary within a large population. We used the estimated population VK/SD parameters to compute the population epidemiological parameters. We simulated 1,000 subjects by drawing 1,000 sets of individual VK/SD parameters from the distributions estimated above, taking into account the correlation between the different distributions. We then simulated the individual viral kinetics, systemic and respiratory symptoms dynamics. We used these simulations to predict how individual infectiousness varies in time. We present the median and inter-quartile range (Q1-Q3) for the parameters computed from the simulations.

We assumed that infectiousness is a function of viral titre and respiratory symptoms. We adapted Chen *et al*.’s^24^ expression for the production of infectious particles as *J*(*t*) = *V*(*t*)(*b*+*sR*(*t*)), where *b* is the baseline infectiousness in the absence of respiratory symptoms and *s* the slope factor. Indeed, infectious particles can also be produced during normal breathing^3^. *R(t)* and *V(t)* are the individual respiratory symptom score and viral titre at time *t* predicted by the within-host model.

For symptomatic subjects, we computed the incubation period as the time from inoculation to reach a total symptom score (i.e. the sum of both respiratory and systemic symptom score) of 1, 2, 3 or 4 and the duration of the symptomatic phase as the time during which the total symptom score is above 1, 2, 3 or 4.

## Results

### Description of the experimental inoculation data

56 volunteers were included in the control arm of the 5 studies of antiviral drug trial and received only placebo treatment. 12 of these subjects did not have a virus-positive sample on any occasion after the challenge and were therefore excluded from the analysis as uninfected. Of the 44 subjects shedding influenza virus, 9 did not report systemic symptoms on any occasion after the challenge and 4 of these did not report respiratory symptoms either; these 4 subjects were therefore considered as asymptomatic. The peak for total symptom (i.e. the sum of systemic and respiratory symptoms scores) had a median score of 6 and ranged between 0 and 20, for a scale ranging between 0 and 24.

**Figure 2:**
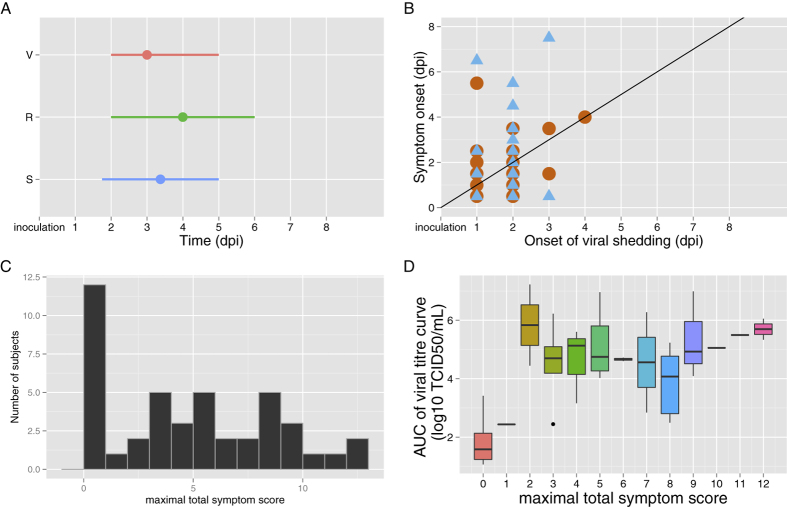
(**A**) Average observed dynamics for viral load (V), respiratory symptoms (R) and systemic symptoms (S). Each segment starts at the median onset of V, R or S and ends when V, R or S is undetectable or not reported. The dot represents the average time of maximal value, tmax. (**B**) Relationship between the symptom onset (orange dot for respiratory symptoms, blue triangle for systemic symptoms) and the onset of viral shedding. Viral shedding onset is defined as the time between inoculation and the first sample above the limit of detection. (**C**) Distribution of the maximal total symptom scores. (**D**) Distribution of AUCV (the area under the viral titre curve) depending on the maximal total symptom scores.

Time courses of virus titres and symptoms scores are summarized in Fig. 2A,B and Table 1. The distribution of the maximal total symptom score in Fig. 2C. Figure 2D shows that on average the area under viral titre curve (AUCV) is similar when the maximal total symptom score is above 2 and is above 3-fold higher than in patients with maximal total symptom score ≤1. For subject with maximal total symptom score ≤1, the median AUCV is 1.58 log_10_ TCID_50_/mL (Q1-Q3: 1.24–2.14) whereas for subjects with maximal total symptom score above 2, the median AUCV is 4.76 log_10_ TCID_50_/mL (Q1-Q3: 4.09–5.52).

### Viral kinetics/symptoms dynamics: Parameter estimates and fits

**Figure 3:**
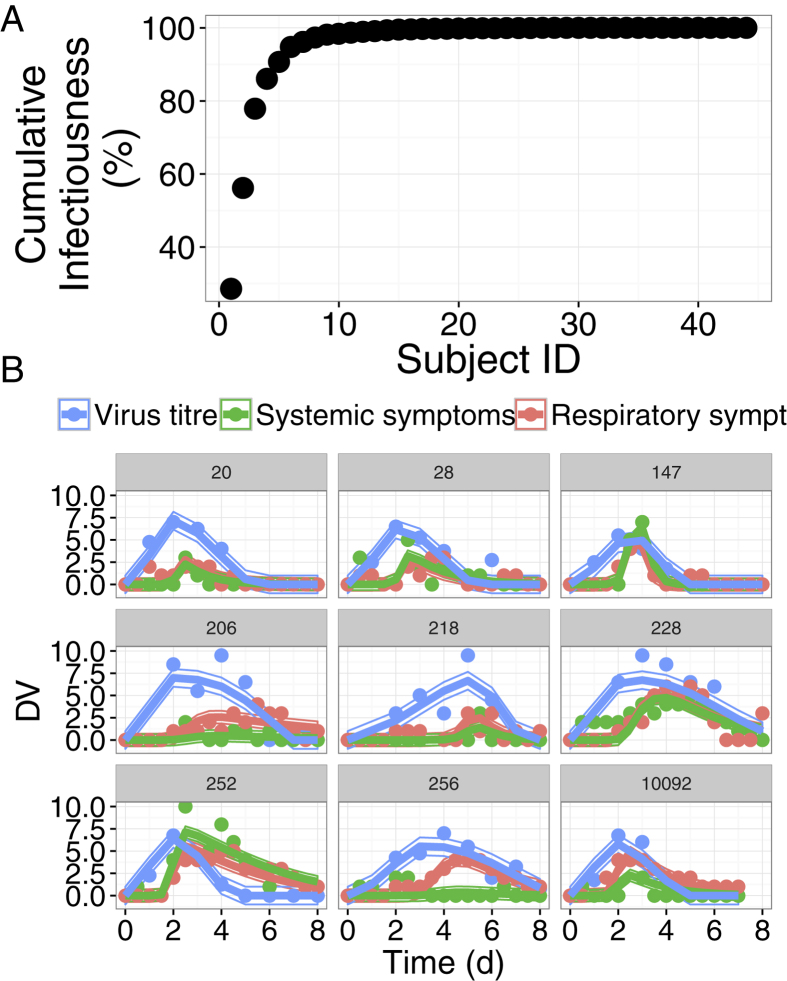
Infectiousness. (**A**) Cumulative infectiousness (percent) for subjects (N = 44) ordered from highest to lowest estimated values of infectiousness. (**B**) Individual fits for the viral titre (blue), systemic symptoms (green) and respiratory symptoms (red) for the 9 most infectious subjects.

**Figure 4:**
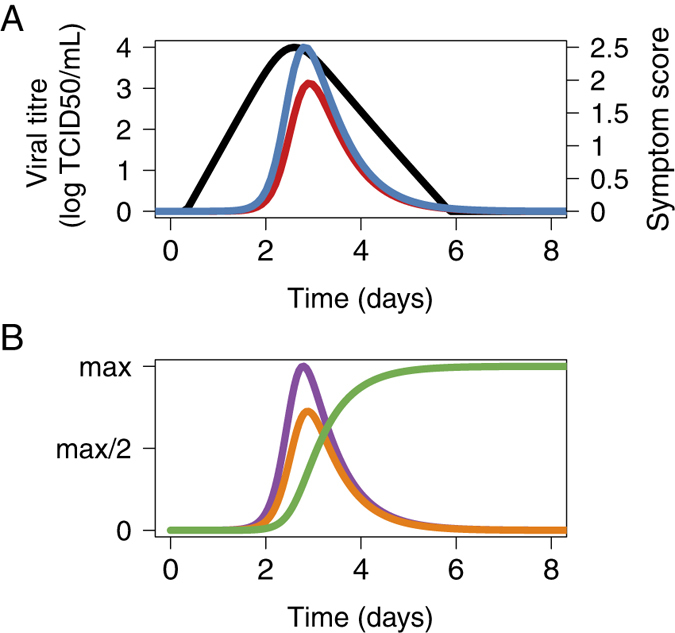
Influenza infection dynamic curves. (**A**) Population fits for VK (black), respiratory symptoms (blue) and systemic symptoms (red). (**B**) Average predictions for local cytokines (purple line), systemic cytokines (orange line) and cytotoxic activity (green) line. Cytokine dynamics were scaled to show the relative proportions of local and systemic cytokines.

Guided by the practical identifiability analysis, we fixed three parameters, *V*_*0*_, *δ* and *c* (Supplementary Information, Fig. S1). Parameters estimates are presented in Table 2. The model provides good fits to virus titre, systemic symptom score and respiratory symptom score (Fig. 3B and S2 A–C) and the goodness of fit plots are satisfactory (Fig. S3). The model predicts that the average virus titre increases sharply to a peak at 2.5 dpi and is then cleared at ~4.8 dpi (Fig. 4A). The local and systemic cytokines levels are predicted to increase until 2.8 dpi and to be cleared at ~10 dpi. Finally, the model predicts that the cytotoxic activity increases slowly until ~10 dpi (Fig. 4B).

Population parameters estimates are presented in Table 2 and individual parameter estimates in Table S1. Most parameters are accurately estimated with a relative standard error (i.e. the ratio of the standard error divided by the average value) <40%. All estimated parameters except *p*, the virus production rate, show inter-individual variability >100%.

On average, the subjects shedding more virus have more symptoms. Indeed, the area under the viral titre curve (AUCV) and the area under the total symptom curve (AUCS) are positively correlated (Spearman’s rank correlation: 0.52, p-value = 0.0003) and the maximal total symptom score and the maximal viral titre are also positively correlated (Spearman’s rank correlation: 0.64, p-value < 0.0001). This shows that viral titre and total symptom score are correlated.

### Natural history parameters

#### Asymptomatic subjects

From the simulations of 1000 subjects, we predict that 30.1 to 67.4% of subjects are asymptomatic or paucisymptomatic, when we set the maximal symptom score threshold between 1 and 4. For the symptomatic subjects, the incubation period lasts on average 1.9 to 2.4 days and subjects exhibit symptoms for an average of 1.5 to 2.4 days, depending on the threshold (Table 3). Symptomatic subjects are 7.4- to 7.8-fold (Wilcoxon test, p-value < 0.0001, for all thresholds) more infectious than asymptomatic subjects. We also find that all parameters are significantly different between symptomatic and asymptomatic. For a maximal total symptom threshold set to 2, infectivity, *β*, is 2.3-fold higher; virus production rate, *p*, 2.1-fold higher, effect of pro-inflammatory cytokines on virus production, *ψ*, 2.9-fold higher, cytotoxicity, *η*, 5.5-fold smaller, local cytokine production rate, *ρ*, 2.4-fold higher, local cytokine clearance, *α*_*L*_, 3.3-fold smaller, systemic cytokines production, *γ*, 2.5-fold smaller and systemic cytokine clearance, *α*_*S*_, 3.1-fold higher in symptomatic than in asymptomatic. The increased cytotoxicity induces a rapid halt in the virus life-cycle, which reduces the subsequent inflammation responsible for symptoms.

The peak of infectiousness occurs 1.5 to 2.2 days later in asymptomatic subjects than in symptomatic subjects.

#### Highly infectious subjects

We predict from our model fits that a limited number of subjects are producing the great majority of infectious particles. Indeed, 5/44 and 7/44 shedding subjects were responsible for 90 and 95% of infectiousness, respectively (Fig. 3A). We tested several models for the infectiousness, with different values for *b* and *s.* These subjects were the most infectious with the different values of *b* and *s* (Fig. S4).

From the 1000 simulated subjects, we predict that 10.9% and 18.2% of the subjects are responsible for 90 and 95% of the total population infectiousness, respectively. Interestingly, our model also predicts that 19% and 20% of these most infectious subjects have symptom score ≤2 and could therefore be considered as asymptomatic. This raises the question of the identification of the most infectious subjects.

Four parameters are significantly (p < 0.0001, Spearman correlation) correlated with infectiousness (i.e. AUC of *J(t)*): the virus production rate, *p*, the cytotoxic activity, *η*, local cytokine clearance rate, *α*_*L*_, and local cytokine production rate, *γ*.

## Discussion

Understanding the temporal dynamics of influenza infectiousness and the factors driving it is relevant to understanding influenza epidemiology and designing effective mitigation measures. The parameter estimates and predictions from our model are broadly consistent with previous findings. Asymptomatic infections have been estimated to account for 30–77% of seasonal influenza cases^19^,^25^,^26^. The wide range of estimates can be explain by the threshold used to detect symptomatic subjects. Indeed we predict that depending on the threshold used, the proportion of asymptomatic subjects in a given sample varies between 30.1 and 67.4%. Most of the estimated parameters are 2.1 to 5.5 fold different between symptomatic and asymptomatic subjects, suggesting that symptoms and infectiousness are associated with complex inflammatory mechanisms. Other host and virus factors can also play a role such as the age or virus strain^27^. Children are more prone to be infected than any age group during seasonal influenza epidemics and to develop severe case or complications^28^ whereas the majority of deaths due to influenza are observed in elderly in developed countries^29^.

Our predicted incubation period of ~2.0 days is close to that found by Lessler *et al*.^30^ who estimated the median influenza A incubation period is at 1.4 days (Q1-Q3: 1.1–1.9 days) from experimental and observational studies. Our predicted duration of symptomatic phase of 1.5–2.4 days is shorter than previously published duration of illness of 4.5–5.0 days in A/H1N1 experimentally infected patients^25^.

Using our model to predict infectiousness, we show high heterogeneity in infectiousness. Indeed, our model predicts that 10.9% and 18.2% of the infected subjects are responsible for 90% and 95% of infectious particles produced, respectively. This heterogeneity in infectiousness adds another level of heterogeneity for influenza transmission, in addition to population structure^31^ and contacts^32^.

Matthews *et al*.^1^ showed that control measures targeting high shedders could be effective strategies to reduce *E. coli* O157 transmission in cattle. Whereas in *E. coli* O157, there is a bimodal distribution of shedding rates^33^,^34^, for other infections such as Salmonella, this is not the case, although shedding rates are highly heterogeneous^35^. We did not identify a bimodal distribution for infectiousness produced in the present work (see Fig. 4A). However, the high degree of heterogeneity of viral shedding implies that targeting the most infectious subjects would have disproportionate benefits. We also predict that, even though on average symptom score is associated with infectiousness, up to 20% of the most infectious subjects are asymptomatic or paucisymptomatic. Consequently, this suggest that highly infectious subjects could remain undetected and would therefore be difficult to target for mitigation measures.

Our study has two main limitations. First the VK and SD data come from experimental infections with a single viral strain of a homogenous population of healthy volunteers with low influenza antibody titres. The applicability of our model to natural infection in adults depends on the pathogenicity of the viral strain as well as pre-existing immunity associated with past influenza exposure and vaccination. Moreover, increased attack rate of influenza among children may reflect their higher susceptibility and infectiousness^36^,^37^. To describe the variability of shedding patterns in the general population, it would therefore be necessary to adapt the model to take into account how the VK parameters changes with age. Second, in our model, the different pro-inflammatory cytokines (interferons, interleukins, TNF) are represented by a single variable, as we did not have data to distinguish them. With the addition of such data, we could provide more refined models for the interaction between the virus and the host and possibly identify more precisely the biomarkers associated with increased infectiousness. If informative biomarkers were identified, highly infectious subjects could be preferentially targeted for interventions. Second, symptom scores were self-reported and data describing thoroughly the number of coughs or sneezes were not available. Particles count and size distribution can vary substantially for different respiratory symptoms and several respiratory symptoms can occur simultaneously in influenza infected subjects. Therefore, to thoroughly describe the impact of the different respiratory symptoms, a daily monitoring of particles production would be necessary. As this information was unavailable in the present study, we used the average respiratory symptom score to predict infectiousness.

In summary, we have developed a new model combining infection and symptom dynamics and used unique data describing the time course of infection and infectiousness and their variability in healthy adults. Our predictions suggest a high degree of heterogeneity in virus shedding by infected subjects. Our work supports targeting mitigation measures at highly infectious individuals to efficiently reduce transmission. Effectiveness of public health interventions would depend on accurate identification of highly infectious subjects and on how quickly control measures can be applied.

## Additional Information

**How to cite this article**: Canini, L. *et al*. Heterogeneous shedding of influenza by human subjects and its implications for epidemiology and control. *Sci. Rep.* **6**, 38749; doi: 10.1038/srep38749 (2016).

**Publisher's note:** Springer Nature remains neutral with regard to jurisdictional claims in published maps and institutional affiliations.

## Supplementary Material

Supplementary Information

## Figures and Tables

**Table 1:** Dynamics of observed viral titre, respiratory symptoms and systemic symptoms.

	Onset (dpi)	End (dpi)	Time of peak (dpi)	Peak value	Number of subjects
Viral titre	2.0 [1.0–2.0]	5.0 [4.0–7.0]	3.0 [2.0 −3.5]	4.6* [3.2–5.8]	44
Respiratory symptoms	2.0 [1.0–2.5]	6.0 [4.5–8.0]	4.0 [3.0–5.5]	3.0 [2.0–4.0]	40
Systemic symptoms	1.8 [0.5–2.5]	5.0 [3.4–6.1]	3.4 [2.5–5.1]	3.0 [1.8–4.3]	35

Results are presented as median and interquartile range [IQR]. Dpi: days post infection. ^*^in log_10_ TCID_50_/mL.

**Table 2:** Viral kinetics and symptoms dynamics parameter estimates.

Parameter	Description	Unit	Population estimate (rse %)	IIV % (rse %)
*V*_0_	Initial viral load	TCID50/mL	0.5 (−)	—
*β*	Infectivity rate	(TCID50/mL)^−1^ d^−1^	1.4*10^−8^ (26)	140 (15)
*δ*	Infected cell mortality rate	d^−1^	0.5 (−)	—
*p*	Virus production rate	(TCID50/mL). d^−1^. Cell^−1^	10.4 (14)	72 (16)
*σ*	Conversion factor from infectious virus into TCID_50_/mL	TCID_50_/mL. Cell^−1^	0.69 (−)	—
*ψ*	Local cytokine effect	F^−1^	8.1 (42)	243 (13)
*η*	Cytotoxic activity	—	221 (32)	188 (13)
*c*	Free virus clearance rate	d^−1^	1.0 (−)	—
*α*_*L*_	Local cytokine clearance rate	F d^−1^	1.56 (20)	117 (13)
*α*_*S*_	Systemic cytokine clearance rate	d^−1^	11.0 (34)	118 (28)
*γ*	Local cytokine production rate	F. Cell^−1^. d^−1^	2.2*10^-5^ (80)	497 (12)
*ρ*	Systemic cytokine production rate	F^−1^	8.19 (38)	149 (24)

Population estimate indicates the average value of the fixed effect, for each parameter. Inter-individual variability (IIV) indicates the average value of the random effect, for each parameter. The relative standard error (rse) is the ratio of the standard error divided by the average value and is computed for each population estimate and IIV. The symbol ‘–’ is used when parameters were fixed. d indicates days. The correlation parameters are shown in the Table S2. The additive error terms are a_1_ = 0.971 log_10_ TCID_50_/mL for viral titre, a_2_ = 0.65 for systemic symptom score and a_3_ = 0.71 for respiratory symptom score.

**Table 3:** Proportion of asymptomatic or paucisymptomatic, incubation period and duration of symptomatic phase in 1,000 simulated subjects.

Threshold	Proportion of asymptomatic or paucisymptomatic (%)	Incubation period (days) [Q1-Q3]	Duration of symptomatic phase (days) [Q1-Q3]
1	30.1	2.0 [1.4–2.8]	2.4 [1.4–4.5]
2	45.0	2.0 [1.5–2.8]	1.9 [1.0–3.6]
3	57.6	2.4 [1.7–4.2]	1.7 [0.9–3.3]
4	67.4	1.9 [1.4–2.6]	1.5 [0.8–3.1]

Parameters were drawn from the correlated distribution of individual parameter. The threshold of total symptom score has been set to 1, 2, 3 or 4. The total symptom score scale ranges from 0 to 24.
