# Supplementary material for: Heterogeneous shedding of influenza by human subjects and its implications for epidemiology and control
Source: Sci Rep. 2016 Dec 14;6:38749. doi: 10.1038/srep38749 (PMC5155248; doi:10.1038/srep38749)
Supplement: Supplementary Information [file srep38749-s1.pdf]

Heterogeneous shedding of influenza by human subjects and its implications for epidemiology and control

Laetitia Canini[1*], Mark EJ Woolhouse[1], Taronna R. Maines[2], Fabrice Carrat[3,4,5]

[1] Centre for Immunity, Infection & Evolution, University of Edinburgh, UK

[2] Influenza Division, National Center for Immunization and Respiratory Disease, Centers for Disease Control and Prevention, Atlanta, Georgia, USA

[3] INSERM, UMR_S 1136, Institut Pierre Louis d'Epidémiologie et de Santé Publique, Paris, France

[4] Sorbonne Universités, UPMC Univ Paris 06, UMR_S 1136, Institut Pierre Louis d'Epidémiologie et de Santé Publique, Paris, France

[5] Assistance Publique Hôpitaux de Paris, Hôpital Saint Antoine, Paris, France

Supplementary material

Text S1: Supplementary methods

Description of the nonlinear mixed effect models.

Nonlinear mixed effect models (or the population approach) allows a description of population characteristics (fixed effect) as well as the inter-individual variability (IIV, random effect)[1]. In this method, a function f describing the variables being modeled, e.g., the viral load, depends nonlinearly on θ_i, a vector of the p parameters of subject i. A vector ξ_i representing the times at which samples are collected from subject i, $\xi_i = (t_{i1}; t_{i2}; \ldots; t_{in})$, is also considered. The statistical model for subject i is then given by:

$$y_i = f(\theta_i;\xi_i) + \varepsilon_i(a + bf(\theta_i;\xi_i))$$

where y_i is a vector with n_i observations of subject i, with i varying from 1 to N, ε_i is the vector of the residual errors which is the part of the observations unexplained by the model f. It is assumed that the errors ε_i are independent from one observation to another and that their distribution is Gaussian $\varepsilon_i \sim N(0; I_{ni})$, where I_{ni} is an identity matrix of dimension n_i. a and b are two parameters characterizing the error model variance. To fit the data, we used an additive error model: $y = f + a\varepsilon$, where y is the observed response, f is the model function, a is the additive error term, and the error, ε, is normally distributed following N(0,1).

In nonlinear mixed effect models, the model f is common to all the subjects, but the vector of parameters θ_i for subject i may vary from one subject to another. The inter-individual variability is modeled with the vector of random effect parameters η_i.

The vector of parameters θ_i for the subject i can then be expressed as a second-level model which links with the function g, the vector of fixed effect parameters β common for all subject and the vector of random effects b_i specific for subject i: $\theta_i = g(\beta;\eta_i)$. The vector of random effect is assumed to follow a Gaussian distribution $\eta_i \sim N(0;\ \Omega)$, η_i and ε_i are assumed to be independent for subject i and $\eta_i|\varepsilon_i$ is assumed independent from one subject to another. Ω is the matrix of random effect variance and covariances. The function g is here an exponential model. The vector of parameters is hence written as $\theta = \beta e^{\eta_i}$.

Parameter estimation and statistical methods. Population parameter estimates and inter-individual variability (IIV) estimates were obtained using a maximum-likelihood method implemented in MONOLIX version 4.3.3 (http://software.monolix.org), which uses the stochastic approximation expectation-approximation (SAEM) algorithm[2] to estimate population parameters. The VK model was fitted to $\log_{10}$ viral load. HCV genotype, cohort and patient type (treatment naïve vs. non-responder) were included as covariates in the model

to study their effect on the PK/VK parameters. Individual parameters were estimated using the empirical Bayes method[3].

The model was simultaneously fitted to VK, systemic and respiratory symptom data since simultaneous fitting has been shown to provide more precise estimates [4,5]. Parameters estimated were b, p, ψ, η, α, α_S, γ and ρ. For each parameter, we report the population estimates and their relative standard errors, as well as the inter-individual variability in percent with its relative standard error (Table 1).

Individual parameters were estimated using the empirical Bayes method [3] and are presented in Tables S1. In the following we present only the significant results.

<u>Dimension analysis</u>

The ODEs compartments are the following:
T: unit = cell
I: unit = cell
V: unit=TCID50/mL
F_L and F_S: unit= arbitrarily set to F (this represent the cytokines levels)
N: cytotoxic activity: unit= Cell^{-1}.d^{-1}

Cell.d^{-1}=beta.cell.TCID50/mL
Cell.d^{-1}=beta.cell.TCID50/mL – delta.cell – eta.Cell. Cell^{-1}.d^{-1}
TCID50/mL.d^{-1}=p.Cell/(1+psi.F) – s.TCID50/mL - sigma .beta.cell.TCID50/mL
F.d^{-1}=gamma.Cell – alpha$_L$.F
F.d^{-1}= rho.F - alpha$_S$.F
Cell^{-1}.d^{-2} = k.F

We can then deduce that:
beta = (TCID50/mL)$^{-1}$d^{-1}
delta= d^{-1}
eta = Cell
psi = F^{-1}
c = d^{-1}
sigma = Cell^{-1}.TCID$_{50}$/mL
p = TCID50/mL.d^{-1}.Cell^{-1}
gamma = F.Cell^{-1}.d^{-1}
alpha$_L$= d^{-1}
rho=d^{-1}
alpha$_S$=d^{-1}

$k = F^{-1}.Cell^{-1}.d^{-2}$

Practical identifiability results

To choose the values for these parameters, we re-estimated the parameters for every combination of the following V_0=0.1, 0.5 and 1.0 $TCID_{50}$/mL, δ=0.5, 1.0 and 2.0 d^{-1} and c=1.0, 3.0 and 10.0 d^{-1}. These values are in the same range as the parameter estimates obtained in previous human vivo studies [6-8]. We chose the set that provided the smallest Bayesian information criteria (BIC), where V_0=0.5 $TCID_{50}$/mL, δ=0.5 d^{-1} and c=1.0 d^{-1} (Fig. S1). We found that four parameters, p, ζ, γ_S, α_S did not vary significantly between the different fits and that infectivity rate, β, tended to be lower whereas production of local cytokines (α_L) and diffusion of cytokines to circulation (ρ) tended to be higher with lower virus clearance, c. The effect of cytokines (ψ) tended to be higher with lower infected cell mortality rate (δ) (Fig. S1).

Supplementary Information references

1 Karlsson, M. & Sheiner, L. The importance of modeling interoccasion variability in population pharmacokinetic analyses. *J Pharmacokinet Pharmacodyn* **21**, 735-750 (1993).

2 Kuhn, E. & Lavielle, M. Maximum likelihood estimation in nonlinear mixed effects models. *Comput Stat Data Anal* **49**, 1020-1021-1038 (2005).

3 Pinheiro, J. & Bates, D. (New York: Springer Verlag, 2000).

4 Zhang, L., Beal, S. L. & Sheiner, L. B. Simultaneous vs. sequential analysis for population PK/PD data I: Best-case performance. *J Pharmacokinet Pharmacodyn* **30**, 387-404, doi:10.1023/B:JOPA.0000012998.04442.1f (2003).

5 Savic, R. & Karlsson, M. Importance of shrinkage in empirical Bayes estimates for diagnostics: problems and solutions. *AAPS J* **11**, 558-569 (2009).

6 Baccam, P., Beauchemin, C., Macken, C. A., Hayden, F. G. & Perelson, A. S. Kinetics of influenza A virus infection in humans. *Journal of virology* **80**, 7590-7599, doi:10.1128/JVI.01623-05 (2006).

7 Canini, L. & Carrat, F. Population modeling of influenza A/H1N1 virus kinetics and symptom dynamics. *Journal of virology* **85**, 2764-2770 (2011).

8 Hancioglu, B., Swigon, D. & Clermont, G. A dynamical model of human immune response to influenza A virus infection. *Journal of theoretical biology* **246**, 70-86, doi:10.1016/j.jtbi.2006.12.015 (2007).

Table S1: Parameter estimates (mode) for individual patients.

Individual parameters are Empirical Bayes Estimates

ID	**Infectivity rate** β **[(TCID50/mL)$^{-1}$ d^{-1}] x10^{-8}**	**Virus production rate** **P** **[(TCID50/mL)$^{-1}$ d^{-1}Cell^{-1}]**	**Local cytokines effect** ψ [F^{-1}]	**Cytotoxic activity** η **[-]**	**Local cytokines clearance rate** α_L [Fd^{-1}]	**Systemic cytokines production rate** ρ **[F^{-1}]**	**Systemic cytokines production rate** α_s **[d^{-1}]**	**Local cytokines production rate** γ **[F.Cell^{-1}.d^{-1}]**
1	2.61	5.00	1.53	59.74	4.63	3.02	2.12	$3.93\ 10^{-4}$
2	8.96	6.87	63.64	101.47	0.83	1.74	18.64	$1.28\ 10^{-7}$
3	0.96	20.30	35.67	604.34	0.40	19.57	43.39	$2.86\ 10^{-6}$
4	1.78	10.36	3.32	112.05	1.85	32.15	6.65	$7.61\ 10^{-6}$
5	3.69	13.88	14.83	637.89	1.11	34.16	44.46	$1.16\ 10^{-8}$
6	2.01	15.75	14.31	424.87	0.75	43.15	35.55	$1.16\ 10^{-7}$
7	0.22	9.48	0.49	1923.90	5.62	5.74	3.91	0.0170
8	0.97	1.91	0.15	563.47	9.11	0.64	1.25	$4.42\ 10^{-4}$
9	1.55	11.56	25.69	166.77	0.69	8.33	11.61	$8.12\ 10^{-6}$
10	0.25	10.58	0.62	1595.80	5.22	6.90	4.18	0.0165
11	0.26	8.76	0.50	1730.40	5.64	4.82	3.62	0.0156
12	1.94	6.19	3.62	178.75	2.36	4.05	5.62	$2.08\ 10^{-5}$
13	3.53	13.33	236.52	17.64	0.34	13.57	23.91	$1.52\ 10^{-7}$
14	0.25	8.13	0.51	1856.00	5.13	4.44	4.25	0.00772
15	1.61	11.85	119.30	5.75	0.54	5.05	9.23	$1.54\ 10^{-4}$
16	0.90	13.45	10.43	139.38	1.23	21.06	13.91	$1.60\ 10^{-5}$
17	0.62	6.56	1.22	247.99	5.23	2.89	2.41	0.00603
18	0.30	7.84	0.42	2191.80	5.74	3.59	3.72	0.0121
19	0.57	10.95	1.41	1686.30	3.13	7.98	7.74	$4.40\ 10^{-4}$
20	4.56	2.38	1.83	145.08	3.27	0.71	3.01	$4.11\ 10^{-6}$
21	1.00	9.75	18.00	158.38	0.95	3.25	12.78	$8.60\ 10^{-5}$
22	4.46	21.25	168.61	271.98	0.43	5.65	42.17	$2.60\ 10^{-6}$

23	1.03	27.30	47.57	68.73	0.83	32.01	30.23	$6.38\ 10^{-5}$
24	4.87	7.37	2.47	52.75	4.26	14.62	9.45	$1.99\ 10^{-6}$
25	3.27	9.02	140.15	117.79	0.43	1.32	19.33	$2.36\ 10^{-6}$
26	0.25	79.53	26.54	1140.60	0.70	437.68	76.25	$4.50\ 10^{-5}$
27	10.21	10.09	230.35	127.84	0.19	8.05	52.65	$7.43\ 10^{-10}$
28	0.30	12.84	1.73	972.28	2.74	21.15	13.69	$1.10\ 10^{-4}$
29	8.06	3.66	3.02	222.50	1.43	5.28	10.80	$9.71\ 10^{-9}$
30	2.86	10.05	41.55	3.85	2.26	17.37	8.06	$3.32\ 10^{-6}$
31	14.64	7.91	54.94	6.85	0.51	24.12	26.14	$2.50\ 10^{-9}$
32	2.77	16.23	27.71	40.58	0.42	69.12	41.36	$1.18\ 10^{-7}$
33	7.50	5.27	39.62	130.83	0.57	1.63	24.98	$3.21\ 10^{-8}$
34	0.31	14.23	0.68	585.35	6.13	16.38	3.98	0.0324
35	0.68	5.29	0.97	693.93	4.08	1.81	2.92	0.00138
36	0.43	9.98	0.70	1670.80	4.67	7.05	4.87	0.00292
37	1.51	11.83	4.76	160.13	2.38	19.40	15.67	$8.98\ 10^{-6}$
38	2.54	9.07	8.82	65.12	1.14	12.69	12.41	$3.11\ 10^{-6}$
39	4.94	16.83	103.35	10.62	0.87	11.65	24.15	$7.32\ 10^{-6}$
40	1.16	25.67	24.92	218.02	1.18	30.56	21.72	$4.92\ 10^{-5}$
41	2.07	18.01	34.40	218.05	0.71	21.18	58.77	$4.94\ 10^{-7}$
42	0.38	4.72	1.26	264.29	3.88	1.25	3.42	0.00472
43	1.51	15.90	208.25	49.76	0.89	2.24	10.04	$3.50\ 10^{-4}$
44	3.36	14.64	89.13	26.93	1.34	7.87	4.54	$5.06\ 10^{-5}$

Table S2: Estimated correlation matrix of the random effects. The relative standard error (rse) is the ratio of the standard error divided by the average value and is computed for each population estimate and IIV.

	β	p	ψ	η	α_L	ρ	α_S
p	-0.25<br>(80)						
ψ	0.54<br>(32)	0.52<br>(31)					
η	-0.66<br>(21)	-0.06<br>(349)	-0.65<br>(18)				
α_L	-0.50<br>(33)	-0.48<br>(33)	-0.91<br>(5)	0.48<br>(32)			
ρ	-0.15<br>(208)	0.76<br>(28)	0.21<br>(128)	-0.12<br>(216)	-0.28<br>(94)		
α_S	0.27<br>(121)	0.71<br>(37)	0.73<br>(31)	-0.25<br>(117)	-0.82<br>(22)	0.59<br>(35)	
γ	-0.82<br>(9)	-0.05<br>(414)	-0.63<br>(20)	0.51<br>(29)	0.70<br>(14)	-0.25<br>(105)	-0.64<br>(37)

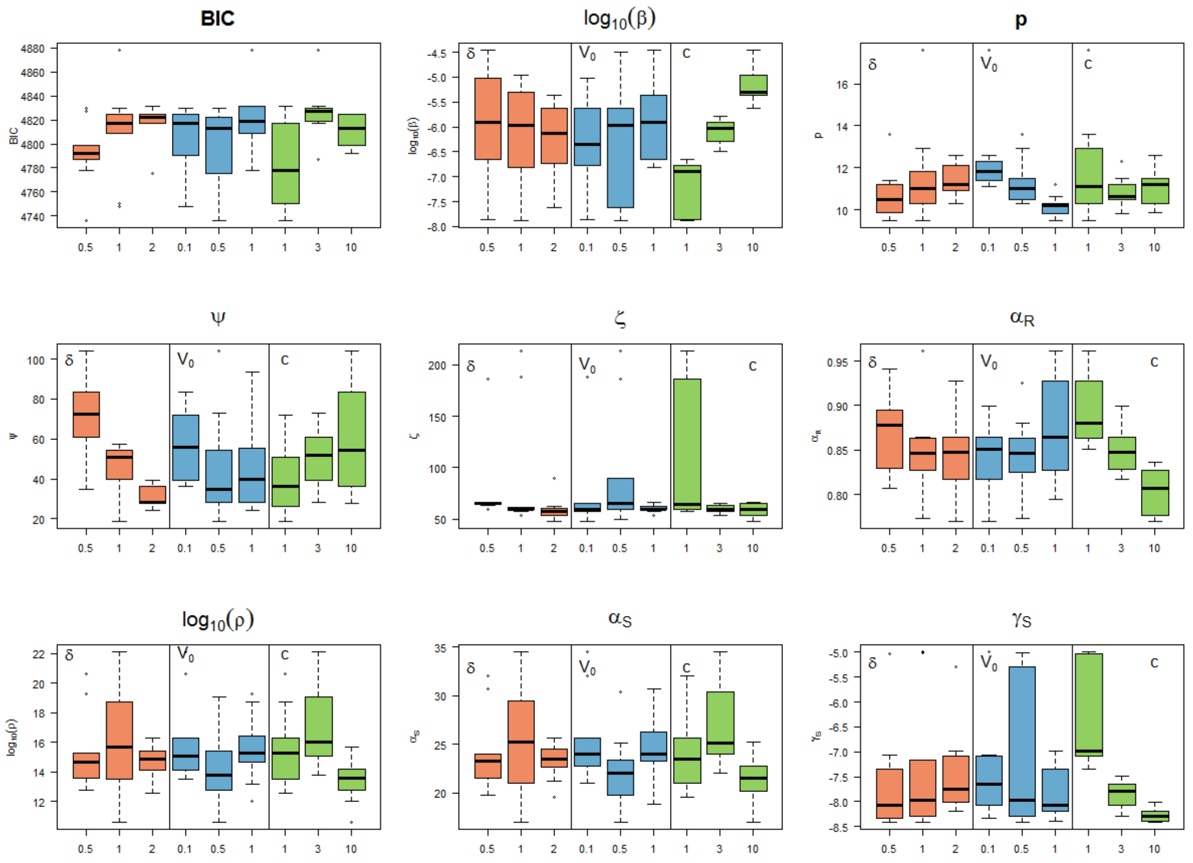

Figure S1. Boxplots of BIC and parameter estimates obtained after fitting the model for different values of δ (orange), V_0 (blue) and c (green). We re-estimated the model parameters for different fixed values of δ, V_0 and c. We tested our model for δ=1.0, 3.0 and 10.0 d^{-1}, for V_0=0.1, 0.5 and 1 log_{10} $TCID_{50}$/mL and for c=0.1, 0.5 and 1.0 d^{-1}. We represent here the BIC as well as the population parameters distribution depending on the value of the fixed parameters.

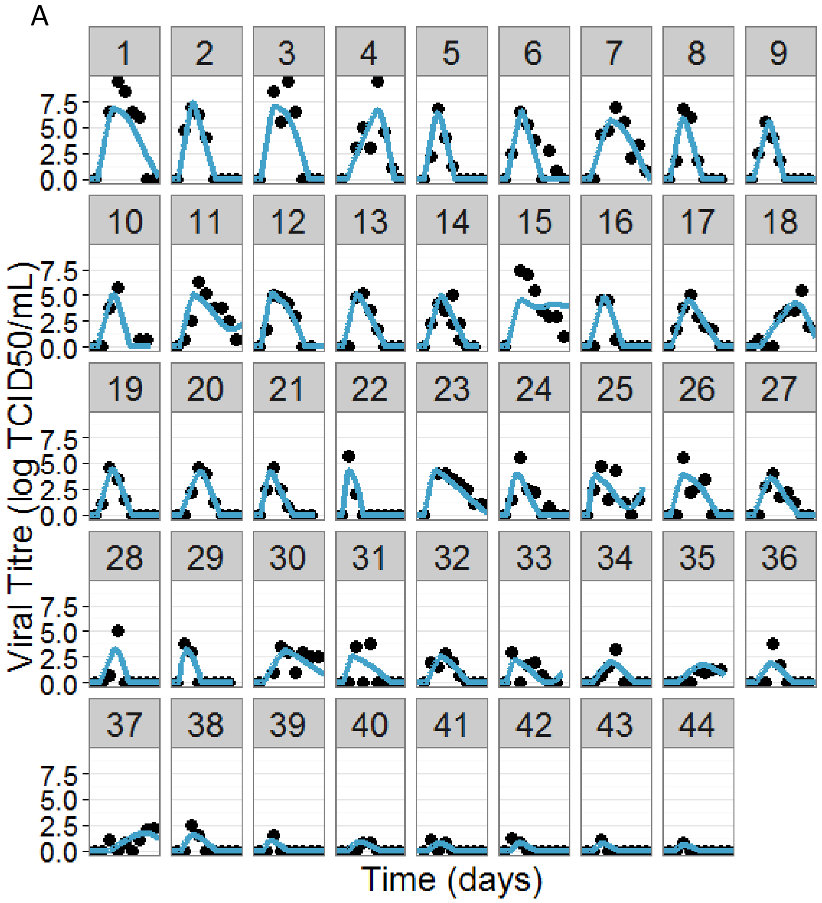
A
Viral Titre (log TCID50/mL)
Time (days)

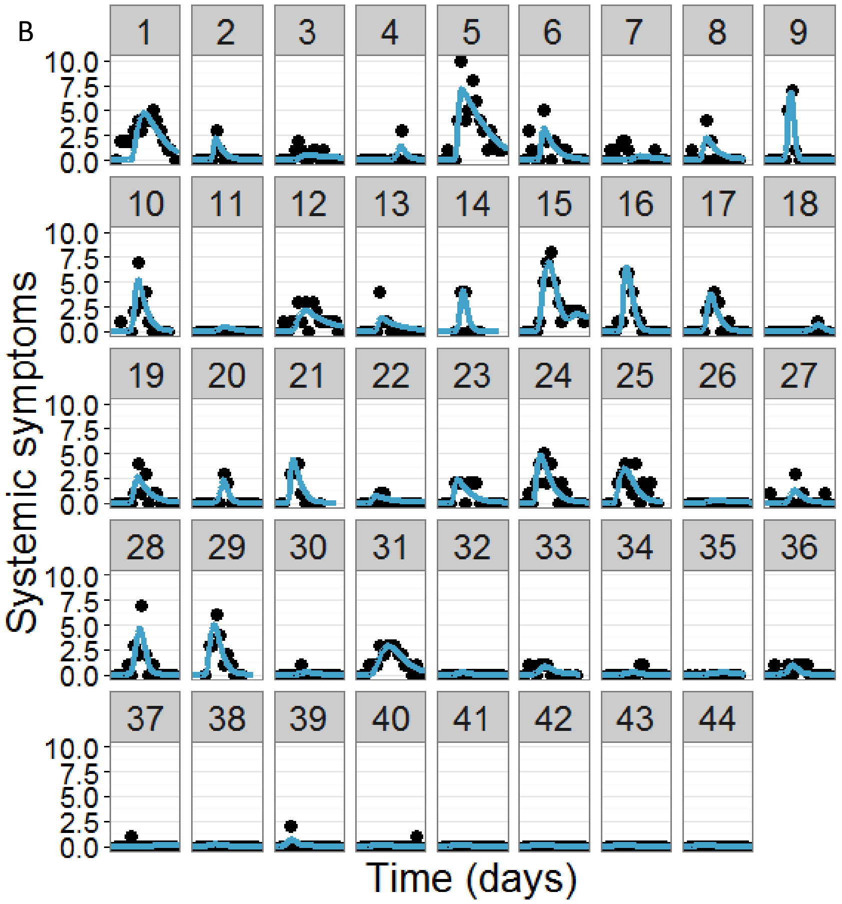
B
Systemic symptoms
Time (days)

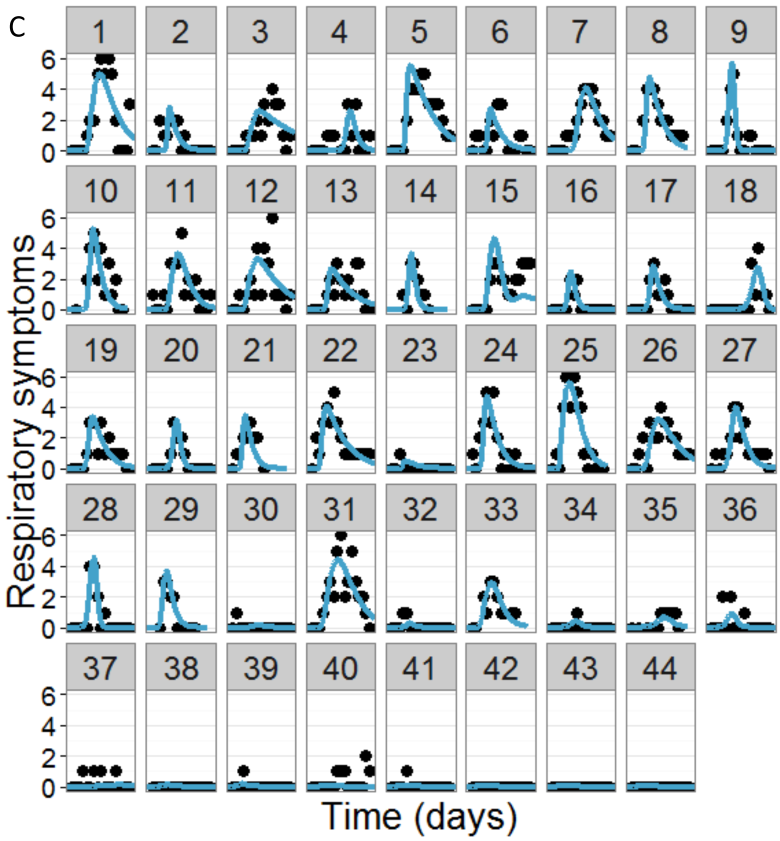

Figure S2. Individual fits (blue line) to observation (black dots) for viral titre (panel A), systemic symptoms (panel B) and respiratory symptoms (panel C)

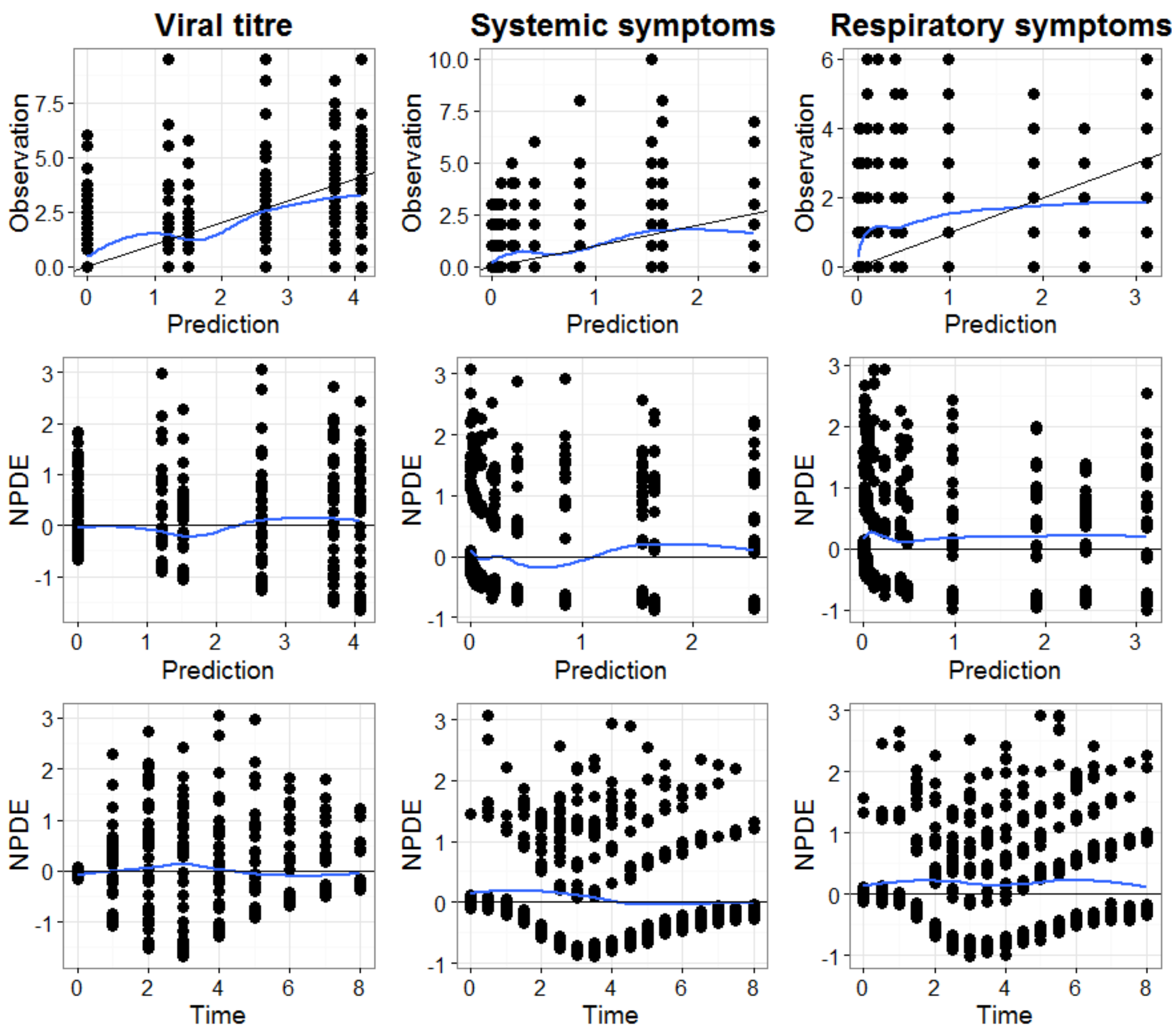

Figure S3. Goodness of fit plots for viral titre (left), systemic symptoms (centre) and respiratory symptoms (right). The upper panels represent the observations vs. predictions, the black line is the line of identity, the blue line the spline and show that the model fits well the data. The central panels represent the Normalised prediction distribution errors (NPDE) vs. the predictions and show no trends in the distribution of the residual depending on the predictions and the lower panels represent the NPDE vs. time and show no trends in the distribution of the residual depending on the sample time. Overall these goodness of fit plots suggest that the model fits well the data.

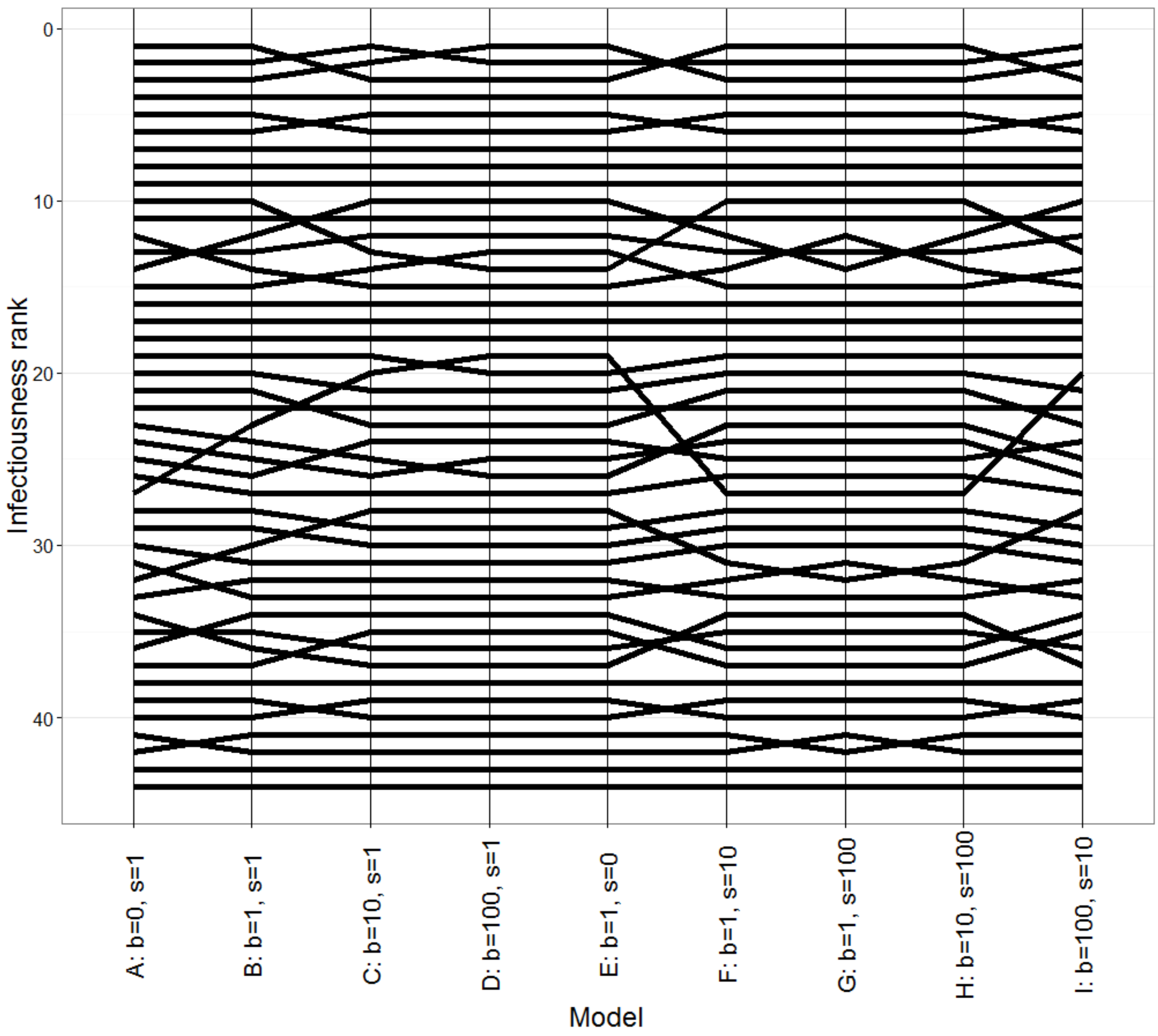

Figure S4. Effect of parameters *b* and *s* on the infectiousness rank. Infectiousness is computed as the area under the curve of $J(t) = V(t)\big(b + sR(t)\big)$. Rank #1 is for the most infectious subject. Each line shows the evolution of a subject infectiousness rank for the different scenarios tested.

Monolix code:

```
DESCRIPTION: Influenza viral kinetic, systemic and respiratory symptoms
dynamics

INPUT:
parameter = {V0, beta, delta, p, psi, eta, c, sigma, alpha, gamma_R,
alphasys, gamma_S}      ; Parameters to estimate

EQUATION:
odeType = stiff

;;;;;;;;;;;;;;;;;;;;;;;;;;;;;;;;;;;;;;;;
; compute the initial values
t0 = 0
Tt_0 = 4e8  ; Fixing initial number of target cells to 4x10^8
I_0 = 0
V_0 = V0    ; Estimating initial viral titre
F_0 = 0
F_s = 0
N_0 = 0
;;;;;;;;;;;;;;;;;;;;;;;;;;;;;;;;;;;;;;;;;

; System of ODE
ddt_Tt = -beta*Tt*V                      ; Target cells
ddt_I = beta*Tt*V - delta*I -eta*I*N      ; Infected cells
ddt_V = p/(1+psi*F)*I - c*V -sigma*beta*Tt*V    ; Free virus
ddt_F = gamma_S*I - alpha*F         ; Local pro-inflammatory cytokines
ddt_Fs = gamma_R*F-alphasys*Fs      ; Systemic pro-inflammatory cytokines
ddt_N = Fs                          ; Cytotoxicity

LVL =max(log10(V),0)    ; Log-transformed viral titre
logistS =12*(Fs/(1+Fs)) ; Transformation of systemic pro-inflammatory
                              cytokines levels into systemic symptom score
logistR = 12*(F/(1+F))  ; Transformation of local pro-inflammatory
                        cytokines levels into respiratory symptom score

OUTPUT:
output = {LVL, logistS, logistR}    ; Fitting simultaneously viral titre
                                    and symptoms scores
```
